# Examining the Impact of a Behavior Modification Program on Disease Prevention Behaviors among Individuals at Risk of Diabetes: A Quasi-Experimental Investigation

**DOI:** 10.3390/ejihpe14070131

**Published:** 2024-07-06

**Authors:** Thanatkorn Phudphad, Supat Teravecharoenchai, Panit Khemtong, Wanich Suksatan

**Affiliations:** 1 Department of Public Health, Faculty of Liberal Arts, Krirk University, Bangkok 10220, Thailand; kornthanatkorn@gmail.com (T.P.); fedupnk@ku.ac.th (P.K.); 2 Midwest Health Professionals, Saint Louis, MO 63119, USA

**Keywords:** behavior modification intervention, blood sugar levels, diabetes, exercise, health behaviors, social media, type 2 diabetes prevention

## Abstract

This study aimed to develop and test a behavior modification intervention to prevent type 2 diabetes (T2DM) among at-risk individuals. The primary goal was to compare diabetes prevention behaviors and fasting blood sugar levels between the intervention and comparison groups. This study utilizes a quasi-experimental design to develop a behavior modification intervention for preventing diabetes. It involves two groups, each with pre- and post-intervention assessments, comprising 60 at-risk individuals equally divided into intervention and comparison groups. The 8-week intervention includes components like risk assessment, dietary skill enhancement, exercise, stress management, and social media platforms (data recording training, follow-up visits, reminders, and visual aids). Data collection includes self-administered questionnaires and blood sugar level measurements. Statistical analysis involved paired *t*-tests for within-group comparisons and independent *t*-tests for between-group differences. The findings showed that the intervention group achieved significantly higher average scores in nutrition, exercise, and stress management, and had significantly lower average blood sugar levels compared to the comparison group. These results suggest that healthcare providers and policymakers should develop community health programs and public health policies that incorporate integrative care, leverage social media platforms, and foster collaboration with other health professionals to improve outcomes for individuals at risk of T2DM.

## 1. Introduction

Type 2 diabetes mellitus (T2DM), a chronic non-communicable disease, currently represents a significant global public health challenge, with its prevalence steadily increasing each year [1]. There were 537 million individuals diagnosed with diabetes worldwide in 2021, a figure projected to rise to 643 million by 2030 and 783 million by 2045 [2]. In the Pacific region, as of 2017, Thailand reported 6.1 million individuals with diabetes, ranking fourth after China, Indonesia, and Japan [2]. The incidence of diabetes in Thailand is continually rising, with an estimated 300,000 new cases annually and 3.2 million individuals currently registered in the Ministry of Public Health’s system. Global health expenditure related to diabetes was estimated to be USD 966 billion in 2021 and is projected to increase to USD 1045 billion by 2045 [1]. Moreover, diabetes remains a primary contributor to the onset of various other non-communicable diseases (NCDs), including heart disease, stroke, hypertension, and kidney disease [3].

Given the incurable nature of diabetes and its potential for genetic transmission, prevention in individuals at risk is more crucial than ever. The long latency period before symptoms appear causes many at-risk individuals to remain unaware and fail to manage their condition early on [4]. Although the Ministry of Public Health of Thailand has implemented the Chronic Care Model as a guideline for managing care for people at risk for diabetes, it has been found that the results still lack consistency in practice as a model to promote concrete behavior change [5]. Consequently, the number of at-risk individuals and diabetic patients continues to rise [6].

The increasing trend in T2DM represents a significant public health problem that requires urgent intervention to reduce future incidence rates [7,8]. The most effective way to control and prevent diabetes is through lifestyle changes, particularly for those at risk. Emphasizing the prevention of diabetes development in high-risk groups is crucial, as individuals at high risk can delay or even avoid the onset of the disease by modifying their lifestyle behaviors (therapeutic lifestyle changes) [9]. Early detection and lifestyle interventions, such as regular physical activity, choosing appropriate foods, consuming vegetables and fruits high in fiber, and avoiding sweet, fatty, and salty foods, are essential [10]. Additionally, learning to manage stress and maintaining a positive outlook can significantly reduce the incidence of diabetes [11].

A comprehensive review of the literature and previous research on behavior modification programs for individuals at risk for diabetes and T2DM populations, both in Thailand [12,13,14] and internationally [15,16,17], indicates that foreign countries employ a diverse array of health behavior modification programs. These programs aim to impart self-management knowledge and elevate awareness regarding personal healthcare. In similar contexts, the effects of behavior modification programs on the exercise behavior of high-risk individuals with T2DM have shown that the average exercise behavior score of the intervention group was significantly higher than that of the comparison group after the experiment [13,14,18]. The findings from these studies suggest that the most effective strategy to address T2DM involves preventing its onset in at-risk groups by enhancing their knowledge and perceptions, thereby promoting positive changes in self-care behaviors [6].

In the context of Ratchaburi Province, Thailand, there is a notably high rate of diabetes patients. The rate of diabetes per 100,000 people for the fiscal years 2019, 2020, and 2021 is 638.15, 585.04, and 596.68, respectively. These figures indicate a likely increase in the rate of new diabetes cases per 100,000 people [19]. Additionally, Mueang Ratchaburi District, the fifth most populous subdistrict in Ratchaburi Province, has been actively screening for diabetes risk groups. Reports summarizing the results of diabetes risk screenings for individuals aged 35 years and over from fiscal years 2019 to 2021 indicate a continuous increase in the number of people at risk, with percentages of 0.81%, 33.43%, and 45.91%, respectively [19].

In response to this trend, we have developed a behavior modification model to prevent diabetes in groups at risk for T2DM, utilizing the Model of Self-Regulation to design activity plans that facilitate appropriate health behavior changes in these at-risk groups. The Model of Self-Regulation suggests that individuals’ perceptions of their illness influence their coping strategies, which in turn affect health outcomes like glycemic control and quality of life [20]. Key components of this model include illness perceptions, general coping strategies, and specific strategies like adherence to treatment and health behaviors. These elements are essential for sustaining behavior changes and have been incorporated into our behavior modification model developed in this study.

Furthermore, we have incorporated the use of the LINE application to transmit health knowledge and enhance self-care skills related to diet, exercise, stress management, and problem-solving. This includes interactive video calls and sending LINE stickers to provide reminders and follow-ups, which are as effective as home visits and suitable for the current context, where the use of remote media has increased [21,22]. Therefore, this study aims to develop a behavior modification model to prevent diabetes in individuals with T2DM, and these outcomes were assessed before and after the program implementation. This approach is particularly beneficial in the COVID-19 situation, where social distancing reduces the need for home visits and hospital visits, allowing for continuous communication with the health team to address problems.

## 2. Materials and Methods

### 2.1. Research Design

This research employed a quasi-experimental design to evaluate the effectiveness of a behavior modification model for preventing diabetes in high-risk groups. This design was chosen due to several key factors and offers a practical and robust framework for evaluating the effectiveness of our behavior modification program, providing insights that are both applicable and actionable in real-world public health settings.

### 2.2. Setting and Participants

The participants of this study were individuals at risk of diabetes from Mueang Ratchaburi District, Ratchaburi Province, Thailand. The inclusion criteria for the sample group were as follows: individuals at risk for diabetes, both male and female, aged 35 years and above, proficient in reading, writing, and understanding Thai, possessing a smartphone, having a blood sugar level (DTX) of 100–125 mg/dL, being capable of exercise and self-care, and willing to participate in the modification program. Exclusion criteria included a diagnosis of diabetes by a physician, pregnancy, the presence of congenital diseases requiring continuous medication, relocation, requesting withdrawal from the study, or incomplete participation in the study.

The sample size for this study was determined using the G*Power 3.1 program [23]. An alpha significance level was set at 0.05 (α = 0.05), with a power of 0.80 and an effect size of 0.80 [24], resulting in a required sample size of 27 participants per group. To account for potential dropouts, the sample size was increased by 15%, resulting in 30 participants per group. Consequently, the total sample size was 60 participants, divided into two groups, the intervention group and the comparison group, matched to have similar characteristics (matched pair). This study was conducted from September to December 2022.

### 2.3. Research Instruments

The three research instruments utilized to collect data were as follows:

Sociodemographic Data Form: This form consists of multiple-choice and fill-in-the-blank questions covering gender, age, marital status, education level, occupation, sufficiency of income, alcohol consumption, smoking, and receipt of information about diabetes.

Diabetes Prevention Behavior Scale (DPBS): Developed by the researchers through a review of the relevant literature and the Model of Self-Regulation [20], this scale comprises 57 questions divided into three areas: nutrition (30 questions), exercise (17 questions), and stress management (10 questions). For example, one question asks, “Do you read nutrition labels before purchasing food?” The scale employs a 5-level rating system: 1 = never practicing, 2 = rarely practicing, 3 = sometimes practicing, 4 = practicing almost every time, and 5 = practicing regularly. The criteria for interpreting diabetes prevention behavior are as follows: minimal (mean score 1.00–1.50), little (mean score 1.51–2.50), moderate (mean score 2.51–3.50), very high (mean score 3.51–4.50), and highest (mean score 4.51–5.00). This tool was examined by five qualified experts who tested for validity and item-objective congruence (IOC). Validities of nutrition, exercise, and stress management were 0.94, 0.96, and 0.92, respectively. The reliability of this instrument was tried out with 30 samples by finding Cronbach’s alpha coefficient (α–coefficient), which equaled 0.94. The Cronbach’s alpha coefficients in each component were as follows: nutrition questionnaires = 0.92, exercise questionnaire = 0.94, and stress management questionnaires = 0.96.

Health Record Form: This form includes open-ended questions and sections for recording health data and blood sugar levels (Dextrostix: DTX) from fingertip measurements taken after fasting for at least six hours, at two different times. The criteria for interpreting blood sugar levels are as follows [25]: normal (<100 mg/dL), at risk of diabetes (100–125 mg/dL), and diabetes (≥126 mg/dL).

### 2.4. Data Collection

After receiving ethics approval from the research committees, the research team held a meeting to determine the scope of the research and the tools to be used. The researchers coordinated with the Subdistrict Health Promoting Hospital to request permission to collect data, clarifying the objectives and steps involved in conducting the research. The intervention procedure involved a study of the results of the prevention model for type 2 diabetes in individuals at risk. The researchers explained the participation process and obtained signed consent from the participants before implementing the modification model to prevent diabetes in the intervention group. There was no dropout in this study because we used the LINE application, including LINE stickers and messages, to maintain consistent communication with participants. This included sending reminders, motivational messages, and educational content related to diet, exercise, and stress management. Participants received individualized feedback based on their progress, which was designed to be constructive and encouraging, helping them to understand their achievements and areas for improvement. Additionally, we worked with participants to set realistic and achievable goals. Regularly reviewing and adjusting these goals helped maintain their motivation and adherence to the program. Conducted between September and December 2022, the program lasted 8 weeks and included the following activities:

Intervention Group:

Week 1:

- Evaluate diabetes prevention behavior and sugar levels before the experiment (pre-test).
- Organize activities at the Subdistrict Health Promoting Hospital, starting with relationship building and providing knowledge about diabetes. The intervention group participated in self-assessment of their diabetes risk. Activities included learning about eating, exercising, and managing emotions/stress through three activity stations. Participants wrote down goals to achieve within the two months of the program, practiced recording daily information via Google Forms, and set up a LINE group for behavior change communication and sending reminder stickers.

Week 2:

- Organize activities at the Subdistrict Health Promoting Hospital to review knowledge and exchange experiences using models of at-risk individuals with good self-care practices. Small groups were formed to encourage and remind each other to practice daily diabetes prevention behaviors. Group discussions addressed problems and obstacles in practice.
- The intervention group practiced daily diabetes prevention activities and sent information via Google Forms. Encouragement and praise were provided through the LINE group.

Week 3:

- Organize home visits at designated meeting points to exchange experiences, solve problems, and boost morale. Review training on eating, exercise, and emotional management skills for the intervention group to practice daily diabetes prevention behaviors; information sent via Google Forms. Encouragement and praise continued through the LINE group.
- Follow-up visits by phone/LINE to monitor progress, address obstacles, and provide encouragement.

Week 4:

- Organize home visits at designated meeting points to exchange experiences, solve obstacles, and strengthen morale. Review training on eating, exercise, and emotional management skills for the intervention group to practice daily diabetes prevention behaviors; information sent via Google Forms. Encouragement and praise continued through the LINE group.
- Follow-up visits by phone/LINE to monitor progress, address obstacles, and provide encouragement.

Weeks 5–7:

- Organize activities at the Subdistrict Health Promoting Hospital to exchange experiences, solve obstacles, and strengthen morale. Review eating, exercise, and emotional management practices for the intervention group to practice daily diabetes prevention behaviors and send information via Google Forms. Encouragement and praise continued through the LINE group.
- Follow-up visits by phone/LINE to monitor progress, address obstacles, and provide encouragement.

Week 8:

- Organize activities at the Subdistrict Health Promoting Hospital to summarize the results of the activities, provide words of encouragement to the intervention group, and make a commitment to continue correct and sustainable behaviors. Evaluate diabetes prevention behavior and blood sugar levels after the experiment (post-test).

Comparison Group:

Week 1: Personal information and diabetes prevention behavior questionnaires were collected at the Subdistrict Health Promoting Hospital, which took 15–20 min. Participants received 5–10 min of individual general advice on self-care for diabetes and were advised as needed if they had questions.
Weeks 2–7: Participants in the comparison group took care of themselves at home as usual.
Week 8: The researchers repeated the data collection. After completing data collection, the researchers offered an 8-week behavior modification program on disease prevention behaviors to the comparison group.

### 2.5. Data Analysis

The Statistical Package for Social Sciences (SPSS) version 25.0 software was employed for data analysis. Descriptive statistics, including percentages, means, and standard deviations, were used to analyze general data. The normality of the data distribution was assessed using the Kolmogorov–Smirnov test. Independent *t*-tests were utilized to compare the mean scores for nutrition behavior, exercise, stress management, and blood sugar levels between the intervention and comparison groups post-intervention. Paired *t*-tests were conducted to assess differences in mean scores for food consumption behavior, exercise, mood management, and blood sugar levels before and after the intervention within the intervention group. Statistical significance was determined at the 0.05 level (*p*-value <0.05).

## 3. Results

In this study, the intervention and comparison group samples of 60 people had similar characteristics. In the intervention group, the average age was 57.8 years (S.D. = 9.72). The majority were married (70.0%), had a lower primary education level (40.0%), and were generally employed (40.0%). The average monthly income was USD 185.19 (S.D. = 160.04), with 57.1% having enough income to save, 86.7% not drinking alcohol, and 100% not smoking.

In the comparison group, the average age was 51.6 years (S.D. = 5.28). Most were married (56.7%), had a lower primary education level (43.3%), and were occupied with housework (33.3%). The average monthly income was USD 189.52 (S.D. = 125.29), with 53.3% having sufficient income but not enough to save, 96.7% not drinking alcohol, and 100% not smoking, as shown in Table 1.

After participating in the behavior modification program, the intervention group exhibited higher diabetes prevention behaviors in each component and overall compared to before the program. These behaviors were significantly more pronounced than those of the comparison group. The results of comparing the mean values of diabetes prevention behaviors revealed that after participating in the program, the intervention group demonstrated higher diabetes prevention behaviors than before the intervention and significantly higher than the comparison group. Additionally, it was found that the mean blood sugar levels after abstaining from food for 6 h were lower in the intervention group after participating in the program compared to before the program, and significantly lower than those of the comparison group, as shown in Table 2.

## 4. Discussion

The results of this study indicated that the intervention group, after participating in the program, exhibited better overall diabetes prevention behaviors, including each component and blood sugar levels, compared to before participating in the program and significantly better than the comparison group. This improvement can be attributed to the behavior modification model to prevent diabetes, which included demonstrations and practice sessions. The intervention group was able to observe successful behavior modification experiences for diabetes prevention. Practicing eating and exercise habits after the demonstrations allowed the intervention group to achieve their own successes. The program also involved monitoring and supporting activities through a self-directed program combined with the LINE application. The Model of Self-Regulation, based on the Kanfer and Hagerman [20] concept, posits that a person’s behavior cannot be changed by anyone other than themselves and that behavior change relies on motivation, leading individuals to accept and recognize the benefits of such change.

In this study, it was found that most of the intervention group were motivated to engage in the activities because they wanted to avoid suffering from chronic diseases. The intervention group recognized the benefits of dietary changes, exercise, and stress management. Activities included setting weight loss goals to reduce the risk of developing diabetes due to behavioral factors. Participants in the intervention group were asked to analyze the causes of being overweight and their inability to control their weight, and then set short-term and long-term goals that matched their needs. The intervention group received training in various skills, including calculating energy and food portions for each meal, selecting food exchange menus, practicing belly rubbing on a chair, breath-holding practice (prana training), and receiving pamphlets on diabetes prevention to take home and review. This training enabled the participants to understand and accurately implement these practices [15]. They were encouraged to self-regulate by recording their daily food intake, exercise frequency, and stress management each week using Google Forms. Additionally, they conducted self-assessments by comparing their body weight data with their set goals. The experiment found that those who consistently lost weight saw better results, recognized the benefits of the activities, and were motivated to continue participating in the activities.

For cases in the intervention group where the results did not meet the goals, participants received reinforcement and adjusted their goals accordingly. Additionally, the intervention group received support from members of a LINE group established for communication. This group shared various information in the form of text, images, video clips, and stickers. For example, they posted pictures of daily meals to aid weight loss and exercise clips from each team to encourage and follow and provided opportunities to ask questions and give suggestions. The use of the LINE application for communication allowed the intervention group to talk, exchange information, provide reminders, and ask questions about any problems they encountered while performing activities [21]. This constant communication ensured that the intervention group received accurate information, had confidence in continuing their behaviors, and felt encouraged to keep participating in the activities. In addition to verbal praise from LINE group members, messages and stickers were sent to reinforce positive behaviors [26]. In this study, the researcher team supported participants by making home visits and follow-up phone calls to assess the self-regulation progress of the intervention group. This consistent reinforcement helped the intervention group maintain their behaviors throughout the 8-week program, resulting in significant improvements in their eating, exercising, and stress management behaviors.

From the above information, it can be seen that the intervention group improved their eating behavior by controlling food portions according to the specified energy guidelines and developed better eating habits. Weighing themselves weekly and observing weight loss provided encouragement to continue self-regulation and also reminded their partners to self-regulate according to their goals. This is consistent with previous studies [13,14,18], which examined the effects of a self-regulation program on food consumption behaviors in individuals at high risk for diabetes. The results indicated that after participating in the program, the intervention group had significantly higher mean food consumption behaviors compared to the comparison group, with statistical significance at the 0.05 level. Our findings are also consistent with the study in Spain [27], which investigated the effects of a Spanish diabetes self-management program to promote self-efficacy in changing food consumption behavior in individuals with T2DM based on self-efficacy theory. After the experiment, the intervention group showed increases in average scores for self-efficacy in each component, diet, physical activity, and disease control, ranging from 0.5 (95% CI: 0.1 to 0.9) to 0.8 (95% CI: 0.5 to 1.2) [27]. Furthermore, our study aligns with the findings of a meta-analysis from 30 meta-analyses published between 2007 and 2017 aggregating data from 409,185 participants [28]. Their research indicated that after receiving the interventions to promote healthy eating, the intervention groups exhibited better eating habits compared to the comparison group, with statistical significance [28].

In this study, the exercise behavior of the intervention group improved significantly after using the behavior modification model to prevent diabetes, transitioning from a low level to a high level. After the experiment, the intervention group showed an average increase in exercise behavior. This improvement was due to daily practice and self-regulation, with participants recording their exercise each day via Google Forms throughout the 8-week program. The findings of this study are consistent with previous studies in Thailand [13,18], which examined the effects of a self-regulation program on the exercise behavior of high-risk individuals with diabetes. The results indicated that, after the experiment, the average exercise behavior score of the intervention group was significantly higher than that of the comparison group (*p* < 0.05) [13]. Additionally, the results from a previous study showed that the diabetic risk group in the intervention group had a greater increase in the mean exercise behavior score than the comparison group (*p* < 0.001), with the mean score increasing by 0.75 points (95% CI: 0.49, 1.00) [18]. Furthermore, our study corroborates the findings of a previous study conducted in the United States, which demonstrated that the self-regulation strategies with the most significant increase in frequency over time included tracking physical activity, considering one’s environment, rewarding oneself for engaging in physical activity, making physical activity more enjoyable, setting goals, and experimenting with different types of physical activity [28]. These changes were statistically significant at the *p* < 0.001 level.

In terms of stress management behavior, we found that the intervention group improved from a moderate to a high level after using the behavior modification model to prevent diabetes. Additionally, the intervention group had higher stress management behavior scores than before receiving the model, and they were higher than the comparison group. This can be explained by the model’s inclusion of stress management techniques, such as meditation and breath-holding practice (prana training), demonstrated by the researchers. The program also facilitated an exchange of experiences within the group regarding stress management, enabling the intervention group to practice stress management independently. These findings are consistent with the studies in Thailand, which found that after participating in the empower home visiting program using a family tree, the sample group had a higher mean score for stress management and disease prevention behaviors than before participating in the program, with statistical significance at the 0.05 level (*p*-value <0.05) [29]. Clinical outcomes, including blood pressure levels, blood sugar levels, and body mass index, also improved significantly [29]. Similarly, the previous reviews investigated the effectiveness of a health behavior change program to improve adherence to chronic disease medications [30], showing that the intervention group developed stress management skills, resulting in positive perceptions of their experiences. Consequently, the intervention group was able to effectively manage stress [31].

The primary strength of the present study lies in its originality, as it is the first to develop a behavior modification model for individuals at risk of T2DM in rural communities by incorporating the use of the LINE application and Google Forms to transmit health knowledge and enhance self-care skills related to diet, exercise, stress management, and problem-solving. By applying self-regulation theory, the behavior modification model successfully facilitated behavioral changes in eating habits, exercise, and stress management, and led to improved blood sugar levels. These findings underscore the effectiveness of the behavior modification model in preventing T2DM, demonstrating its potential applicability to at-risk groups in similar contexts. The behavior modification model used in this study can be adapted to urban environments where access to healthcare facilities and technology might be more readily available. Urban areas often have diverse populations with varying levels of health literacy and access to resources. By tailoring the intervention to consider these factors, such as leveraging local community centers for in-person workshops or using mobile health (mHealth) technologies to reach a wider audience, the program can effectively address the needs of urban residents. The use of the LINE application in this study highlights the potential for digital tools to support health interventions. In urban and culturally diverse settings, similar platforms that are popular and widely used by the target population can be utilized to maintain communication, provide educational content, and support behavior change. This approach ensures that the intervention is accessible and relevant to the participants’ daily lives. Additionally, to effectively implement and sustain T2DM prevention programs, healthcare providers and policymakers should develop comprehensive training and education modules, implement patient-centered care plans, and utilize technology for continuous engagement and monitoring. They should also establish standardized systems for tracking progress, allocate sufficient funding, and promote community-based interventions. Public awareness campaigns and cross-sector collaboration are crucial, along with integrating diabetes prevention into existing health programs and fostering international cooperation. These steps will enhance the effectiveness of T2DM prevention initiatives and improve health outcomes for at-risk populations.

The effectiveness of the behavior modification intervention to prevent T2DM among at-risk individuals can be significantly influenced by various cultural and contextual factors. Culturally, dietary habits and exercise norms in Thailand may differ from those in other countries, affecting how participants engage with dietary recommendations and physical activity guidelines [32]. For instance, traditional Thai diets rich in rice and certain fats might need specific adaptations in the intervention to align with healthy eating practices. Additionally, cultural beliefs about health and illness can shape participants’ perceptions of T2DM prevention and their willingness to adopt recommended behaviors [3]. Finally, policy and environmental factors, such as government policies promoting healthy lifestyles and the physical environment’s support for physical activity and healthy eating, can significantly impact the intervention’s effectiveness. Supportive policies and a conducive environment can facilitate the adoption of healthy behaviors and sustain the intervention’s benefits.

The present study has several limitations. First, the participants were individuals at risk of diabetes from only one province in Thailand, which may constrain the generalizability of our findings to other populations in different contexts, such as urban communities and nursing homes. Future studies should explore the application of this behavior modification model in various urban and cultural contexts to validate its adaptability and effectiveness. Comparative studies can help identify which modifications are most effective in different settings, further refining the model for broader application. Second, we utilized only the DTX method to assess blood glucose levels after fasting for at least 6–8 h. While this method is suitable for mass screening programs for individuals at risk of diabetes due to its accuracy and rapid results, future studies should employ other standard methods for evaluating blood glucose levels in individuals at risk of diabetes, such as fasting plasma glucose or HbA1c levels. Third, the current study focused on short-term outcomes, necessitating more in-depth investigation into the relationship between demographic data, health behaviors, and clinical outcomes. Future interventional studies should aim to clarify the long-term outcomes of individuals at risk of diabetes with extended interventions. To ensure the sustainability of the intervention, future research should explore ways to integrate it into routine healthcare practices. This might involve training healthcare providers to deliver the intervention during regular check-ups or incorporating it into community health programs. Scalability and adaptability studies are crucial to determine how the intervention can be expanded to larger populations and adapted to different cultural, socioeconomic, and healthcare contexts. The long-term use of technology, such as mobile apps and social media platforms, should be explored to support continuous education, motivation, and monitoring. Fourth, we developed the DPBS and tested it for validity and reliability. However, future studies are needed to further validate this tool to assess diabetes prevention behavior among individuals at risk for T2DM. Fifth, we used a quasi-experimental design to assess the effectiveness of a behavior modification model for T2DM in high-risk groups. This design is prone to internal validity threats like maturation, selection bias, and regression to the mean. The lack of random assignment makes it difficult to attribute observed effects solely to the intervention. Future research should utilize randomized controlled trials (RCTs) to overcome this limitation. Additionally, further studies will be necessary to explore the interactions between social media and evidence-based practice and to formulate institutional policies that benefit patients, clinicians, public health practitioners, and the industry as a whole.

## 5. Conclusions

Diabetes is a chronic non-communicable disease that leads to numerous complications. The current study aimed to develop a behavior modification intervention for preventing T2DM among individuals at risk and to compare diabetes prevention behaviors and fasting blood sugar levels between the intervention and comparison groups. Our findings revealed that the intervention group exhibited statistically significant higher average scores in nutrition, exercise, and stress management compared to the comparison group. Additionally, the intervention group demonstrated significantly lower average blood sugar levels. These results indicate that the behavior modification intervention had a positive impact on the food consumption behavior, exercise habits, and stress management of individuals at risk for diabetes. Additional research is needed to assess the impact of social media on the dissemination of knowledge in clinical practice and to determine whether it significantly enhances patient outcomes. Consequently, health professionals can leverage these findings to implement preventive measures against diabetes among at-risk groups, addressing this significant public health concern effectively.

## Figures and Tables

**Table 1:** Sociodemographic characteristics of the participants.

Demographic Characteristics	Intervention Group		Comparison Group		Total	
	N	%	N	%	N	%
Sex						
Female	30	100.0	29	96.70	59	98.30
Male	0	0.00	1	3.30	1	1.10
Age (years)	Mean ± SD = 57.8 ± 9.72		Mean ± SD = 58.1 ± 11.87		Mean ± SD = 58.04 ± 10.80	
35–40	1	3.30	2	6.60	3	5.00
41–50	7	23.40	4	13.30	11	18.30
51–60	9	30.00	9	30.00	18	30.00
61–70	9	30.00	9	36.70	20	33.30
>70	4	13.30	4	13.30	8	13.40
Marital status						
Single	2	6.70	3	10.00	5	8.30
Married	21	70.00	17	56.70	38	63.30
Divorced/Windowed	7	23.40	10	33.40	17	28.40
Education level						
Primary school	19	63.30	20	66.60	39	65.10
High school or higher	11	36.60	10	33.30	21	34.90
Current Occupation						
Agriculturist	5	16.70	4	13.30	9	15.00
Businessperson/trade	5	16.70	8	26.70	13	21.60
Employment	12	40.0	8	26.70	20	33.40
Unemployment/retired	8	26.7	10	33.30	18	30.00
Income (USD)	Mean ± SD = 185.19 ± 160.04		Mean ± SD = 189.52 ± 125.29		Mean ± SD = 174.69 ± 142.97	
<140	16	57.1	16	53.3	32	53.4
141–285	10	35.8	10	33.4	20	33.3
>286–430	2	6.60	4	13.30	6	10.00
Prefer not to answer	2	6.60	0	0.00	2	3.30
Income sufficiency						
Sufficient and saving	3	10.00	6	20.00	9	15.00
Sufficient without saving	19	53.30	18	60.00	37	61.70
Insufficient	6	30.00	6	20.00	12	20.00
Prefer not to answer	2	6.700	0	0.00	2	3.30
Alcohol drinking history						
Drinking	2	6.70	1	3.30	3	5.00
No drinking	26	86.70	29	96.70	55	91.70
Quit drinking	2	6.70	0	0.00	2	3.30
Smoking history						
No smoking	30	100.00	30	100.00	60	100.00
Received information about T2DM						
Received	29	96.7	27	90.0	56	93.3
No Received	1	3.3	3	10.0	4	6.7

Notes: SD = standard deviation, T2DM = type 2 diabetes mellitus.

**Table 2:** Outcome of a behavior modification program on disease prevention behaviors among individuals at risk of diabetes.

Variables	Intervention Group		Comparison Group		t	*p*-Value
	Mean ± SD	Interpretation	Mean ± SD	Interpretation		
Diabetes Prevention Behaviors					14.13	<0.01
Before	2.81 ± 0.37	Moderate	3.10 ± 0.38	Moderate		
After	4.09 ± 0.39	Good	2.94 ± 0.21	Moderate		
Nutrition					11.57	<0.01
Before	2.38 ± 0.32	Fair	3.26 ± 0.32	Moderate		
After	3.91 ± 0.30	Good	3.10 ± 0.23	Moderate		
Exercise					11.68	<0.01
Before	2.45 ± 0.62	Fair	2.55 ± 0.84	Moderate		
After	4.24 ± 0.73	Good	2.39 ± 0.46	Fair		
Stress management					8.31	<0.01
Before	3.31 ± 0.51	Moderate	3.55 ± 0.39	Good		
After	4.38 ± 0.44	Good	3.41 ± 0.46	Moderate		
Blood sugar level					−1.962	0.05
Before	120.96 ± 24.48	Risk of Diabetes	108.80 ± 19.68	Risk of Diabetes		
After	98.63 ± 13.35	Normal	108.03 ± 22.58	Risk of Diabetes		

## Data Availability

The data sets aside from the research information of this study, are not publicly available due to information that could compromise the research participants’ privacy. However, data may be shared upon request to the authors.
